# The Hot (Invisible?) Hand: Can Time Sequence Patterns of Success/Failure in Sports Be Modeled as Repeated Random Independent Trials?

**DOI:** 10.1371/journal.pone.0024532

**Published:** 2011-10-05

**Authors:** Gur Yaari, Shmuel Eisenmann

**Affiliations:** 1 Department of Pathology, Yale School of Medicine, Yale University, New Haven, Connecticut, United States of America; 2 HIL Applied Medical, Mishor Yamin, Arava, Israel; Humboldt University, Germany

## Abstract

The long lasting debate initiated by Gilovich, Vallone and Tversky in 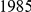 is revisited: does a “hot hand” phenomenon exist in sports? Hereby we come back to one of the cases analyzed by the original study, but with a much larger data set: all free throws taken during five regular seasons (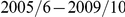) of the National Basketball Association (NBA). Evidence supporting the existence of the “hot hand” phenomenon is provided. However, while statistical traces of this phenomenon are observed in the data, an open question still remains: are these non random patterns a result of “success breeds success” and “failure breeds failure” mechanisms or simply “better” and “worse” periods? Although free throws data is not adequate to answer this question in a definite way, we speculate based on it, that the latter is the dominant cause behind the appearance of the “hot hand” phenomenon in the data.

## Introduction

Current information era, brings with it exciting opportunities for exploring old and new research fields using extensive data sets that are easily accessible nowadays. It allows scientists to explore many topics on a larger scale and in a more precise quantitative way. Sports is a great example of how one can take advantage of large data sets that are available in a digital format and address interesting questions in a variety of contexts (eg. [1]–[6]). Hereby, we study a large data set from the world of basketball: any person who ever watched a basketball match is likely to be familiar with terms like “hot hand”, “on fire”, “in the zone”, “on a roll” etc. These terms are intended to describe the belief that an individual's performance temporarily increases significantly beyond his or her normal rate. The “hot hand” phenomenon has generated a huge interest in the past 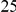 years since Gilovich, Vallone and Tversky [7] published their pioneering paper in Cognitive Psychology. The original motivation of their paper was to study how human subjects misperceive random sequences and tend to attribute non-random patterns to completely random data. For that purpose they analyzed three types of data coming from the world of basketball. They claimed that the observed patterns could have been produced by random as well and hence the fact that people relate “temperature” inspired adjectives to players in different times is connected to the way human beings perceive the random world surrounding them and not to the objective features of reality. Since this provoking work many people have analyzed basketball and other sports looking for evidence of deviations from random patterns (see the reviews of [8], [9] and the website [10]). However, since data is inconclusive in most cases [8] and evidence for clear deviations from random patterns are rare (e.g. [5], [6], [11]), the question of what type of deviation from the random pattern was hardly addressed. In particular, the nature of deviation from the base rate could be attributed to sequential dependence between events (“success breeds success” and “failure breeds failure”) or to non-stationary probability of success (“better” and “worse” periods). The psychology of the player underlining each type of deviation is completely different and in principal could be a very interesting research topic by its own conditioned that such deviation exists.

Since the illuminating work of Wardrop [12], it is clear that if one is interested in the individual level one should not analyze the aggregated data because strange effects due to Simpson's paradox [13] sometimes referred to as “the fallacy of the averages” [14] might change the results dramatically. Later on, Wardrop [15] did find traces of deviation from the repeated independent Bernoulli hypothesis also in the individual level by analyzing 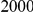 shots of one player (Katie Voigt, who took 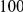 throws each day for 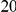 days); the results, however, weren't conclusive as they were based on one individual only, the nature of deviation from the null hypothesis wasn't clear (dependency or non-stationarity) and the setup was controlled and wasn't part of an ongoing basketball game. In the current paper a large amount of data is analyzed: all free-throw sequences extracted from play by play data of five regular seasons of the NBA (data is available at www.nba.com). Indeed, it is shown that there is a dramatic difference in the results if one looks at the aggregated data or the individual level data. Nevertheless, even after taking the effect caused by different individual levels properly into account, we were still able to find “hot hand” traces in the individual level. We argue that this finding is mainly due to time fluctuations of the probability of success and does not necessarily imply that there is a psychological process that influences the performance of a player based on his previous results.

As mentioned above, Wardrop [12] showed that the answer to the question whether a “hot hand” exists in free throws data might be different if one looks at the aggregated level or the individual level. He argued that since the typical basketball fan does not hold different data sets for each player, but rather one aggregated data set for all free throws he had ever seen, the “hot hand” phenomenon that is perceived by the typical fan is real since it is present in the aggregated data set. Wardrop concluded that since data in the individual level didn't show “hot hand” traces, the conclusions of [7] still hold and each individual series of results could be taken as a result of a random number generator of independent Bernoulli trials with a constant probability of success (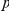). Hereby, we reach a somehow different conclusion and show that the collection of individual sequences is indeed biased toward a hot hand tendency, which might explain Amos Tversky's (who initiated the hot hand research) words: “I've been in a thousand arguments over this topic, won them all, but convinced no one” [8].

## Materials and Methods

### The data

The NBA season is divided into two: regular season and playoffs. In the regular season, each of the 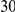 teams plays 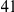 home games and 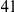 away games, combined to a total of 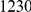 games in a season. For each of these games a “play by play” data is collected and is available through the NBA official website. This data lists all the events that have an influence on the game such as throw attempts, fouls, rebounds, assists, etc. along with the names of the players involved and the time of the event.

There are three types of free throws in a basketball game - all come after a foul was committed. The resulting penalty is a series of either 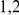 or 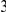 consecutive free throws. A player which is awarded a free throw, gets an uninterrupted attempt to score a basket from a predetermined distance. Since this distance is the same in all the basketball courts and the defending skills of the opposing team and players do not affect the outcome, one is lead to a conclusion that free throws outcome are a measure of the skill of the individual player at that point in time. This makes free throws an excellent candidate to find a “hot hand” provided that such phenomenon exists. Since we were interested only in free throws data the relevant information was extracted from the entire database of five NBA regular seasons (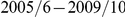). The data set used in our analysis after cleaning it for various items (see next section), is constructed of a total of 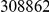 free throw attempts consisting of 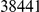 single free throw attempts, 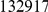 pairs of free throw attempts taken by 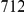 different players and 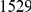 triplets of free throws attempts taken by 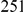 different players from a total 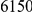 games over 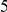 consecutive seasons.

#### Cleaning the data

The first step in analyzing the data was to clean it from all types of errors and inconsistencies:

- The data of 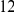 games (out of the 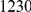 games played in season 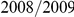) was not complete on the NBA website and therefore was excluded.

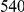
 records of the 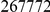 two throws data points had only one entry (the first or second trial). Hence were suspected to be typing errors and were moved into the single throw sets.
- In some cases two players from the same team share the same last name. In most of these cases the player ID or the initial of the first name helps in telling them apart but in several individual cases the data was still ambiguous: in all of these cases we simply ignored this data for the current analysis (sums into 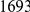 throws out of the 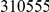 throws exist in the entire data set, 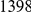 of them were part of two throws sets).

All together, less than 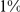 of all data points were ignored for the current analysis. There is no reason to believe that the resulting data is biased in any sense due to the cleaning procedure. The cleaned data is available in Dataset S1.

#### Analyzing the data

As mentioned above there are three types of of free throws sequences: a single attempt, a sequence of two consecutive attempts and a sequence of three consecutive attempts. For all the two attempts sequences of every player, 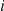, we measured the success rate for the first and second free throws attempts and denoted them by 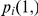 and 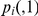 respectively. Throughout this text lower case letters denote single individual properties. Then we measured the conditioned success rate in the second free throw attempt (conditioned the first throw went in/out) denoted by 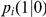 and 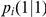 respectively. The average of the players success rates and conditional probabilities were calculated and denoted by an upper case letter using the same notation, 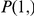, 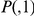, 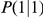 and 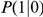. We have also measured the success rates and conditional success rates for the entire data set aggregated over all players and denoted it by 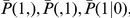 and 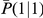. An equivalent procedure was done with the data of the three consecutive free throw attempts.

The results were then tested for statistical significance for two measures:

- Non-stationarity (NS): the change in success rate as the consecutive attempt number increases.
- Conditional probability (CP): the change in success rate of the second attempt for a given results of the previous attempt (for a sequence of three free throw attempts the same was done with the third attempt as well).

Both of these measures can be studied with the aid of the hypergeometric distribution. In order to test the NS one can think of hits as “white balls” and misses as “black balls” and put them all in one urn after labeling them as first or second attempt. Since the null assumption is that there is no systematic deviation in the probability of success between the first and the second attempts, one can sample, without replacement, one half of the total number of throws (first and second attempts combined) and check how many hits (white balls) are in the sample. The null assumption implies that the number of hits in the first or second attempt should be consistent with a random sample from this hypergeometric probability distribution function.

In the case of testing the change in CP one can think of putting all the second attempts as balls in the urn (hits are the “white balls” and misses are the “black balls”). This time the number of balls that are drawn, without replacement, is the number of hits in the first throw. Once again, the null assumption, which states that the result of the second attempt is independent of the result of the first attempt, implies that the number of times one gets hits in both throws will agree with a random sample from this hypergeometric distribution function.

We describe the hypergeometric distribution function with the following parameters: 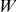 (number of white balls in the urn), 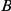 (number of black balls in the urn), 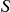 (sample size) and 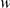 (the number of white balls in the sample) and the data variables by 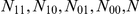 as the number of times the results Hit-Hit, Hit-Miss, Miss-Hit, Miss-Miss, and the total number pairs respectively.

Thus, the formulation of the NS hypergeometric distribution function is,
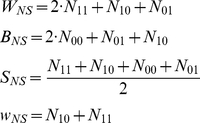
(1)while the formulation of the CP hypergeometric distribution function is,
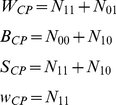
(2)


After calculating these measures, in principal, one can calculate for each individual player, the 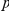 value resulting from an exact Fisher test or an exact Bernard test. Both tests have their own disadvantages in accuracy and more importantly, since the distributions are discrete it is not so easy to analyze the collection of results for all individuals and deduce from it a resulting “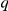 value” or “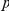 value of the 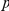 values” [16]. Hence, we decided to take two independent approaches to estimate the probability of obtaining the observed *collection* of result for all individuals just by chance (“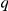 value”).

The first, computationally faster, approach involves estimating a “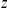 value” for each individual player; the 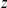 value is the distance (including sign) of the observed value, 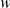, from the expected value, 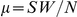, for the hypergeometric distribution in units of its standard deviation,
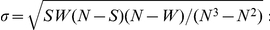


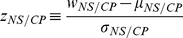
(3)where the subscript is the notations we use to distinguish between the two measures defined above (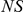 for non-stationarity and 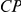 for conditional probability).

When calculating for the aggregated data the total number of free throw attempts is large enough and the distribution of 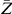 can be approximated well by a normal distribution with zero mean and a variance of 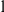, from which the resulting 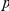 value can be extracted. In cases where we are interested in the statistical significance of the collection of 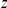 values of the 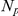 players, one can look at their (the individual 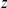
's) mean value: from the definition (eq. 3), assuming the 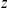
's of the different players are independent, one should expect the mean value to be 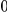 and a variance to be 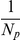. Following this, we define 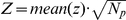 which in turn can use the normal approximation to obtain a “
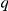
” value by
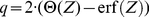
(4)where 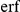 is the Gauss error function and 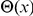 is 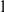 for 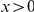 and 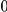 elsewhere. A positive value means “hot hand” while a negative number indicates “cold hand”. This approach is fast but rely on a normal approximation.

A more accurate, though computationally intensive, approach, is permutation approach: first, we reshuffle the second throws of each individual. After reshuffling, we calculate the 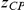 value for each individual for the reshuffled data and record the mean 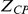 value for this reshuffled realization. Repeating this procedure many times results in a collection of mean 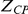 values (one for each realization/reshuffle), each corresponds to independent second throws from the first ones. The last step in order to estimate the correspondent 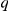 value is to rank the results of the random reshuffles and see what fraction of these show larger values than the actual observed value from the original data. This fraction times two (since we are calculating a two tailed test) is an estimate of the 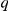 value. In the current analysis we have made 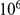 such realizations for each player in each season.

## Results

We start by verifying two observations already pointed out in [12], but here they are observed on a much larger data set which allows for detecting better more subtle effects: 1. Aggregation of data over different players may skew and even reverse the results. and 2. An increase in the success rate with the number of throws attempts (NS). Following these two points, we describe the main results reported here: 3. Even after taking the two previous effects properly into account there is still a statistically significance correlation between the results of consecutive free throw attempts. In our notation 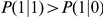, an increase in the conditional probability (CP), which is usually referred to as a “hot hand”. We take, then, yet another step and propose that: 4. The increase in the conditional probability is due to time fluctuations in the probability of success rather than a causal connection between the results of consecutive throws.

### Effect of aggregation

A simple statistics artifact of aggregation, sometimes referred to as “Simpson's Paradox” [13], [14] or the “fallacy of the averages” [17], can produce macroscopic biased patterns out of completely random elements. Although Robert Wardrop [12] observed this effect in a smaller data set he studied (one season of one team - the Boston Celtics), it is instructive to show the presence of this effect in the current data set as it is much more noticeable here and may serve as an intermediate step for a better understanding of the effects caused by time variations of the probability of success. In order to illustrate the effect, let us look at the throw sequences data of two individual players: Dwight Howard and Kevin Martin from the 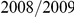 NBA season (summarized in Table 1). While the individual 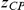 values (see eq. 3 in method section) of both players for conditional probabilities have negative values: 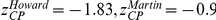, which indicates “anti-correlation” for these players, the combined data and the corresponding 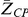 value shows the opposite trend (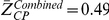), suggesting the combined data shows “hot hand”. According to [12], this might be the hot hand that the typical basketball fan perceives as he/she cannot remember all the individual sequences for each of the players separately but rather one long combined sequence. As is evident from the data presented above, Wardrop argued that observing a hot hand for the data aggregated over all players doesn't necessarily imply that the individual sequences themselves will present such a pattern (see also Supporting Information S1 for some examples of individual players values).

**Table 1:** An example of the Simpson's paradox.

Kevin Martin		
	2nd-Miss	2nd-Hit
1st-Miss	1	35
1st-Hit	25	159

Dwight Howard		
	2nd-Miss	2nd-Hit
1st-Miss	59	102
1st-Hit	89	127

	Both combined	
	2nd-Miss	2nd-Hit
1st-Miss	60	137
1st-Hit	114	286

The individual tables for Kevin Martin and for Dwight Howard (season 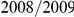) give negative values for 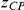 while the aggregated table of both players yields a positive value for 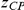.

Yet another demonstration for this kind of bias due to aggregation is presented in the Supporting Information S1 where we show that if one takes a distribution of individual probabilities of success each of which produces an uncorrelated random sequence (that corresponds to the distribution of the different players success rates), the pattern of the aggregated data shows significant correlations in the data (i.e. a “hot hand”) although each individual sequence is uncorrelated by construction.

**Figure 1:**
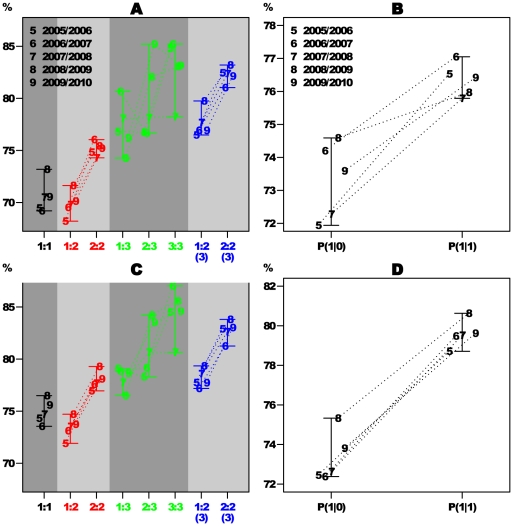
The two trends observed in the data. Panels **a** and **c**(individual and aggregated levels respectively) show how the chances of hitting a free throw increase with the number of throws taken in a row (until a set of three throws). This increase is evident in both individual and aggregated levels (**a** and **c** respectively). The last two values of the 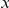 axis represent sets of two throws taken only by individuals who had at least one three throws set. These values resembles the values of the first two throws in a three throws set. Panels **b** and **d** show the success rates of the second throw in a two throws sequence following a success/failure in the first throw. These panel shows a major finding of the current paper: “hot hand” statistical traces - success rates in the second throw are higher when the throw attempt followed a success in the first attempt rather than a failure. In this case as well, the results are present both in the individual level and in the aggregated level (**b** and **d** respectively).

The results for the aggregated level data are presented at the top part of Tables 2, 3 and in Figure 1 (a,b). The aggregated data is a sum of all free throw attempts by all players for each of the seasons. We see that both 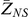 and 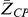 (the 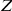 values that correspond to the aggregated data), are positive, suggesting that we are observing the “hot hand”. But, as we learned from the Simpson's Paradox, this alone is not enough to declare that there is a tendency for a player to improve with consecutive free throw attempts (NS) nor that there is a better chance to score after a success in the previous attempt (CP). In order to be able to correctly test for these features we need to look at the data at the individual level: i.e. calculate the level of non-stationarity and conditional probability for each of the players.

**Table 2:** Non stationarity (NS) statistics for each season.

Aggregated data	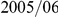	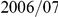	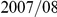	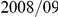	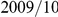
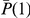	74.32	73.54	74.71	76.49	75.58
Number of throws	7807	8418	7300	7265	7651
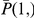	71.91	73.12	73.54	74.70	73.74
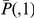	76.95	77.57	77.70	79.29	78.10
Number of throws	27765	27344	26416	25842	25550
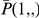	79.07	78.84	77.74	76.51	78.65
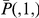	78.29	79.18	80.65	84.23	83.51
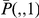	84.50	87.03	80.65	85.57	84.59
Number of throws	258	293	310	298	370
**Individual data**					
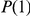	69.47	69.20	70.52	73.19	70.55
Number of individuals	398	410	397	389	397
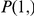	68.21	69.53	69.80	71.63	70.13
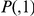	74.83	76.04	74.29	75.43	75.21
Number of individuals	439	443	438	427	429
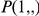	76.82	80.67	78.06	74.26	76.20
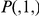	76.73	76.66	78.16	82.06	85.20
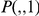	84.79	85.21	78.23	83.06	83.16
Number of individuals	95	112	121	120	132

The upper part of this table (with the ‘ 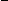 ’ symbols) refers to the success rates in the aggregated data (and number of throws attempts for each of the different free throws types) of the entire regular season (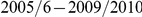). The lower part of the table (without the ‘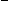 ’ symbols) refers to the mean values of the different players success rates throughout the season. 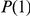 is the percentage of success in a set of one throw attempts. 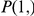 is the same for the first throw out of a two throws set, while 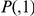 is the percentage of success in the second throw in such a set. 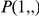 is the percentage of success in the first throw out of a set of three throws attempts and so on.

**Table 3:** Conditional probability (CP) for each season.

Aggregated data	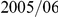	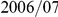	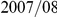	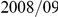	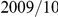
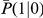	72.45	72.38	72.63	75.33	73.81
Number of records	7800	7350	6990	6537	6709
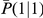	78.71	79.48	79.52	80.63	79.62
Number of records	19965	19994	19426	19305	18841
**Individual data**					
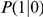	71.94	74.20	72.28	74.59	73.59
Number of individuals	418	424	414	405	408
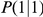	76.53	77.05	75.79	75.97	76.42
Number of individuals	430	435	425	421	422

As in table 1, the upper part of the table (with the ‘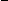 ’ symbols) refers to the aggregated data while the lower part refers to the mean values of the different individual players success rates throughout the season. 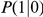 is the success rate in the second throw attempt (out of a two throws set) given that the first one was a miss while 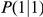 is the success rate in the second attempt given that the first one was a hit.

### Monotonous increase in the probability of success

The success rates of free throws attempts are summarized in Table 1 and shown graphically in Figure 1(a,c). One can see for both the individual (a) and aggregated (c) level data how the mean probability of success increases with the order of throw attempt in the sequence. This effect is evident for the whole period and for each season whether one is looking on sequences of two or three free throw attempts. It is worth noting that the first two attempts from sequences of three free throw attempts have higher mean success rates, this is due to the fact that players who attempt a three point shot usually posses better shooting skills (the blue symbols in figure 1 represents these results). For the case of two free throws attempts, the success rate for the second attempt is increased by at least 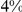 for all the seasons examined with the 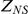 and 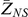 values (see Table 4) for all of the seasons above 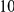 (!). The probability of this happening by chance under the null hypothesis is ridiculously low (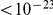) for both the individual and aggregated data, which gives these results a very high level of statistical confidence. Moreover, the fact that both 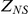 and 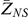 are very close suggests that this tendency of improving the probability for the second shot is relatively a homogeneous feature of the players. The three attempts sequences show the same tendency for the third shot as well but with less statistical significance as there are only 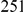 players with 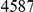 free throws attempts (see Supporting Information S1).

**Table 4:** Statistical significance of the trends observed.

	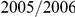	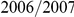	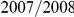	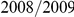	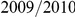
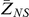	13.62	12.08	11.12	12.39	11.52
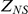	13.48	12.25	10.31	10.9	11.13
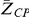	11.13	12.48	11.86	9.15	9.89
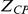	3.71	4.13	4.04	1.87	2.58
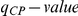	2.1e-04	3.6e-05	5.3e-05	6.1e-02	9.9e-03
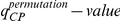	5.2e-04	1.2e-04	1.4e-04	7.6e-02	1.5e-02

As in tables 1,2 the variables with the ‘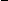 ’ symbol refer to the aggregated data. See text for the definitions of the different measure.

In the left panels (with the red bars) of Figure S1 we plot the histograms for the improvement in success rate for all the individual players and the corresponding 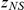 values for each individual player taken from the data for all seasons. The average improvement values span between the limits 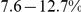 and the median between 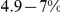 the positive skew from a symmetrically distribution in the individual players level is evident. The histograms of the 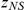 values show a similar skew as well with a means and medians values between 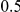 and 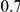, which result, once again, in a negligible 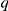 value (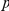 value of the collection of individual 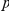 values, see methods) of 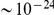 calculated from table 4.

The reasons for this effect could be easily justified as an “alignment gauge” for the hand of the shooter. The time taken by the player until the second attempt also allows for rest and more concentration before the second and third shots are taken. Needless to say that this tendency cannot go on forever and it may be interesting to quantify this feature further using targeted experiments.

### Increase in conditional probability (“Hot Hand”)

Panels b and d of Figure 1 show the success rate of the second free throw attempts conditioned a successful/unsuccessful first attempt for both aggregated and average of the individual players (the data is summarized in Table 3). Over all 5 seasons examined there is a 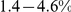 improvement in the second attempt success rate conditioned that the first attempt was good. This data is for the mean of the individual level, eliminating any skew of the results due to aggregation. The histograms for the individual players success rates and corresponding 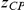 values are presented in the the right blue panels of Figure S1.

As for the statistical significance of this result, one is referred to table 4 to see the resulting 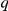 values calculated by the two methods (normal approximation and permutation approach) described in the methods section. One sees that in both methods similar numbers are obtained (which means that the normal distribution approximation is a decent one in this case) and that in most seasons (apart from 2008/2009) the trends we observed are statistically significant (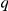 value 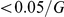, where 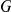 is the number of repeating tests one is performing, 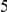 in our case). These 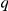 values are much less significant from the aggregated level data. This is attributed to the fact that the players probability of success distribution is broad and the “mean” individual is not a representative player. In three attempts sequences we observed the same trend but with a much lower level of statistical significance due to the low numbers of throws (see Supporting Information S1).

The interpretation of this result is essentially that the results are unlikely to emerge from a collection of uncorrelated sequences each with a constant probability of success and no auto correlation. But statistical significance is borderline in some cases and the question about its' origin remain: does it mean that we found proofs that“success breed success” or can something else explain the observed “hot hand” pattern? Recently, for the change in the conditional probability, similar results were obtained in [6], using different methodology and based on a subset of the data presented hereby (namely, the 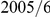 season). Our analysis agree with these results for that season and extend them to a larger period of time.

### “better and worse” periods rather than “success breeds success”

In principle, “better and worse” periods for individual players can cause the same effect as “success breeds success” on the success rate of the second free throw attempt and are hard to separate apart. To illustrate the reasons for this difficulty, two of the simplest descriptions of each possibility are compared:

- “Better” and “worses” periods: switching with random (or constant) periods of time spent in each of the two states where each state is a Bernoulli independent repeated trials with probability of success 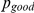 and 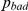.
- Positive/negative one step feedback: the probability of success given the last trial was a success is 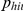 and 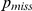 if the previous trial was a failure.

Both options can be viewed as a system where each player behaves essentially like two players sharing the overall sequence with different probabilities of success. This point of view connects back to the first observation (effect of aggregation) and to Supporting Information S1 where it is shown that in this case there will be a systematic deviation between 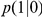 and 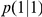 and thus a “hot hand” will be present in the data.

In order to distinguish between the two there is a need for an analysis of the time series of results which will help in identifying a characteristic timescale in which the probability of success changes and then to try and link it to one of the possibilities presented above (see [15] for a comparison of several methods to detect such pattern). However, the studied data set does not allow such analysis since the typical player throws only one set of two throws in a game and it would not be wise to treat free throw attempts from different days as one sequence. Moreover, the free throws happen during a game in which the players experience success and failure in several aspects not captured by the current analysis. We think there will be merit in an experiment that includes many subjects aiming to extend the results in [15] to address this interesting question. In spite of the statistical “hostile” environment for choosing between the two options in this data, it is still possible to come up with two different arguments that support the first possibility (better and worse periods) over the second one (success breeds success and failure breeds failure).

**Figure 2:**
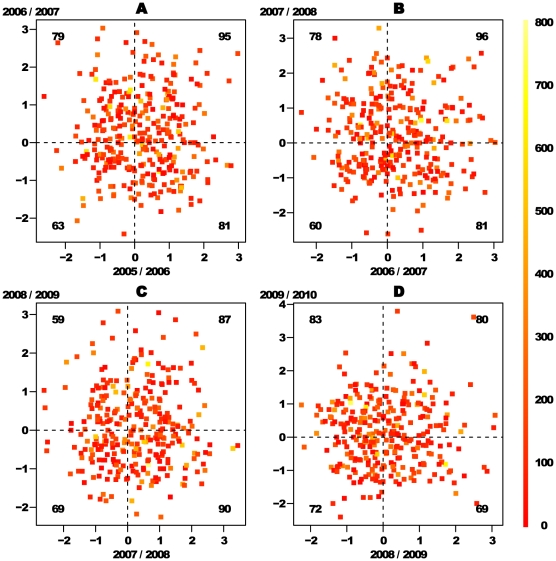
Comparison of the individual 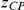 **'s across seasons.** This plot shows the individual 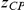 in one season vs. the value of the same individual in the following season (in cases where the player had finite 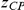 values in both seasons). The color code refers to the total number of two throws sets taken in both seasons. The numbers (in black) in each quartile are the number of observations that fall in each of them. This random pattern of the 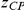 (see text for more quantitative support for this statement) values across seasons suggest that the individual “hot hand” is not a characteristic of the player but rather something that can vary from one season to another for the same individual. This fact leads us to suggest that this phenomenon is caused by whithin-season nonstationary probability of success rather than psychological reasons which are connected to positive/negative feedback loops.

1. ***Common sense***: it will be unrealistic to think that during a year players will not have temporal fluctuations in their objective skills due to various environmental conditions disregarded of their achievements. Some examples are injuries and recovering from them, unrelated psychological crises or improvement in their skills due to hard work.

2. ***Hard wired***: the belief in the positive/negative feedback mechanism is attributed to processes related to the psychology of the player. In particular, it implies that there should be certain players who have a tendency to a “hot hand” and others who have tendencies to a “cold hand” or play neutrally. If such psychological reasons do exist and are hard wired in the players minds, they will dominant the deviation from the null hypothesis. Thus, one expects to be able to see a tendency for each individual to maintain his hot/cold tendency from one season to the other. In figure 2 the 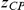 value of each individual player in one season is plotted vs. the value he had for the following year. The numbers of players in each quartile are noted in black and represent the number of players that maintained/flipped their 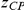 sign (depending on the quartile). One sees that the pattern looks quite random and indeed if one measures Pearson's correlations for these variables the resulting numbers are very close to zero (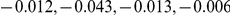 for the four panels ordered by chronological order). In order to prove this point in a more quantitative way we can recruit once again the hypergeometric distribution: in this case, if we denote the four quartiles by 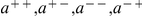, then the mapping to the hypergeometric distribution is
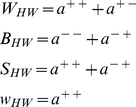
(5)This yiealds the following 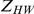 values: 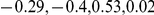 which correspond to the following 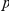 values: 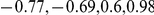. These numbers agree with random reshuffle of the individual 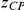 value (which can be thought of as the level of the “hot hand” tendency of each player) from one season to the next one, which implies that a player with “hot” hand in one season has equal chances to have a “cold” hand in the next season. Since here we are not interested in calculating 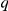 values, we can use also Fisher's exact test for these tables which results in the same kind of results (the resulting 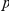 values from Fisher's exact test are: 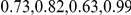).

We conclude that these two points suggest that the observed pattern interpreted as “hot hand” in the analyzed data is in large part a consequence of better and worse periods.

### 4th quarter

It is believed that the last quarter of a basketball game is very different from the other quarters. The game is often interrupted and special tactics apply to this period. An interesting question is if and how the features we have seen so far are affected by this. For that purpose, we have divided the data into two parts: 1) quarters 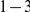 and 2) 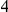
th quarter and overtime and repeated the above analysis. Interestingly, our findings were consistent between these two periods of the game; See Supporting Information S1 for the complete analysis. One more point worth mentioning is that, indeed, as one might expect the percentage of sets of two consecutive free throws taken on the 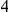
th quarter is significantly larger than 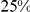 of the total number of sets in the game (except the 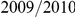 season, in which the deviation is not statistically significant). In addition, the fraction of sets of one free throw is significantly lower than 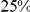 in all seasons. One can connect these observations to the fact that the fouls committed in this quarter are harder and chances of scoring a basket after a foul was committed on the player are lower.

## Discussion

Strong evidence for the existence of a “hot hand” phenomenon in free shots of NBA players were found. More precisely, several statistically nontrivial features of the data were found and can be summed into one concept: heterogeneity. The heterogeneous behavior was found both in “space” (across players) and time (along one season). In particular it has been shown that

- If one looks at the aggregated data he/she is likely to observe patterns that do not necessarily exist at the individual level.
- The probability of success increases with the order of throw attempt in a sequence (NS).
- Even if one looks at each individual sequence separately, “hot hand” patterns are still visible (CP): probability of success following a success is higher than the probability of success following a failure.
- These patterns could have resulted from “better and worse” periods and not necessarily from positive/negative feedback loops.

These statistical features per se are not so surprising when studying performance of human subjects. Nevertheless, due to the intensive debate in the last 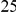 years since the first paper about the “hot hand” phenomenon [7], they can be seen as crucial pieces of information that serve as solid evidences to rule out a stationary randomly independent behavior. In retrospect, it seems like a very long journey to walk through just in order to notice that human subjects have good periods and bad periods and that the time sequence results can not be produced from a binomial independent repeated trials with a constant probability of success. We hope that this work will pave the way for studying the more important questions concerning the “hot hand” phenomenon such as what are the physiological and psychological causes for the changes in the probabilities of success and how do the players and observers perceive these indicators for good and bad periods. In particular, it will be constructive to find new examples and/or stage new experiments that will allow one to measure the time scale in which the good and bad periods alternate. Except [15], the only supported example we have found in the literature that claims that there is a causal connection (dependency) between one trial and the following was from the world of Bowling (see the review by [8]). In that study [5], came to a conclusion that there is a dependency between trials. We re-examined their test for independence and found that the test they have used could have detected nonstationarity rather than dependency. Nevertheless, the Bowling setup seems to be a good place to perform such analysis that will address the question of timescales of good and bad periods as well as what causes the transitions between the two periods.

From the basketball fan/professional perspective, it could be beneficial if the NS result which implies that the probability of success increases with the shot number would be further exploited. To start with, this could be added as one more statistical feature that is calculated and presented throughout basketball matches, but more importantly, one can study further the psychological and physiological reasons behind it and maybe come up with techniques that will help the player to improve the first trial(s) in a sequence of trials.

Although the phenomenon that was studied here is taken from the world of sports, the implications are much more far reaching and should bear in one's mind when analyzing data of any kind. In spite of the fact that it is known for many years that aggregated data (or mean behavior) can deviate significantly from the microscopic dynamics underlying it (e.g. [13], [14], [17]–[20]), it tends to be forgotten in many cases; in particular when referring to *time aggregation*.

The current example of the “hot hand” phenomenon serves as a fascinating one since it is possible to trace back the fundamental reason for the deviations between the observed macro patterns and the underlying micro processes causing them to *heterogeneity* both in *space* and in *time*. It is demonstrated that not only different individuals may have display different characteristics which need to be treated with extra precautions, but also that the “mean behavior” of one individual can be non representative of it's own true *time dependent dynamics*.

## Supporting Information

Figure S1
**Histograms of the individual statistics for the two observed trends for** 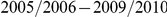 **.** The left, red, panels (A–L) relate to the nonstationary observation (NS): the second throw attempt in a set of two attempts has better success rate than the first one. The left of which (A,C,E,G,I,K) refer to the relative increase of the second throw success rates in units of the success rate of the first (in percentage) while the right panel of which (B,D,F,H,J,L) is a histogram of the 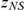 value calculated from the hypergeometric distribution. In both measures infinites might occur, hence the difference in records number. The right, panels (in blue, M–X) show similar measures for the conditional probability (CP): given that the first throw attempt in a set of two went in or out what is the success rate in the second one. The left of which (M,O,Q,S,U,W) refer to the relative difference between the two in units of the percentage of 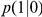 while the right of which (N,P,R,T,V,X) are histograms of the 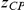 values calculated from the hypergeometric distribution. Although many of the individuals do not show the observed trends, their distribution is unlikely to result from random fluctuations but rather from a real bias toward these trends.(EPS)

Supporting Information S1
**Appendix and supporting figure and tables.** In the appendix we analyze the effect of aggregation on heterogeneous population. The first table lists examples of individual data for the top 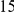 players (ranked by the number of two throws attempts taken) for the 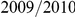 season. The other tables summaries the data for sets of three free throws, and give detailed comparison between the 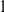
st–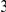
rd quarters and the 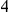
th quarter and overtime.(PDF)

Dataset S1 Data for all free throws taken during 2005/6–2009/10 in the NBA.
(DAT)
