# Supplementary material for: The Hot (Invisible?) Hand: Can Time Sequence Patterns of Success/Failure in Sports Be Modeled as Repeated Random Independent Trials?
Source: PLoS One. 2011 Oct 5;6(10):e24532. doi: 10.1371/journal.pone.0024532 (PMC3187751; doi:10.1371/journal.pone.0024532)
Supplement: Supporting Information S1 — Appendix and supporting figure and tables. In the appendix we analyze the effect of aggregation on heterogeneous population. The first table lists examples of individual data for the top players (ranked by the number of two throws attempts taken) for the season. The other tables summaries the data for sets of three free throws, and give detailed comparison between the st–rd quarters and the th quarter and overtime. (PDF) [file pone.0024532.s002.pdf]

SUPPORTING INFORMATION:
The Hot (Invisible?) Hand: can time sequence patterns of success/failure in sports be modeled as repeated random independent trials?

Gur Yaari[1,*], Shmuel Eisenmann[2]

1 Department of Pathology, Yale School of Medicine, New Haven, CT 06511, USA

2 HIL Applied Medical Ltd., Rotem Industrial Park, Mishor Yamin, Arava, 86800, ISRAEL

∗ **E-mail: gur.yaari@yale.edu**

1 Effect of aggregation on heterogeneous population

Let us consider two players, i and j, each of which throws random independent throws with probability of success equals to p_i or p_j respectively. The two players throw $2*T$ consecutive throws and the probability of success in the $2*t+1$ throw given that the $2*t$ throw went in/out (the average over all t's is denoted by $P_{i/j}(1|1/0)$) is calculated. By construction, each of this time series is not auto correlated and thus

$$P_{i/j}(1|1) = P_{i/j}(1|0) = p_{i/j} \tag{S1}$$

(with errors on the order of $o(\sqrt{\frac{p_{i/j}-p_{i/j}^2}{T}})$). On the other hand, since the aggregated data has half the data of player i and the other half of player j, for long enough series the probability of having a success given that the previous throw went in for the aggregated data is

$$P(1|1) \underset{T_i \to \infty}{\longrightarrow} \frac{p_i^2 + p_j^2}{p_i + p_j} \tag{S2}$$

while the (aggregated) probability of having a success given that the previous throw was a failure is

$$P(1|0) \underset{T_i \to \infty}{\longrightarrow} \frac{(1-p_i)\cdot p_i + (1-p_j)\cdot p_j}{2-p_i-p_j} \tag{S3}$$

These expressions are equal only in the case where $p_i = p_j$ and here lies the key for understanding the paradox. When the probabilities of the different individuals are not the same then the result of the “mean probability” has artificial consequences such as non vanishing time auto-correlation. If one is interested in generalizing this result into a more generic distribution of N players $p_i i \in \{1 \ldots N\}$ the procedure is straight forward. I.e the probability of having a success given that the previous throw was such for the aggregated data will be:

$$P(1|1) = \frac{\sum_{i=1}^{N} \frac{S_i^2}{T_i}}{\sum_{i=1}^{N} S_i} \underset{T_i \to \infty}{\longrightarrow} \frac{\sum_{i=1}^{N} T_i \cdot p_i^2}{\sum_{i=1}^{N} T_i \cdot p_i} = \frac{<p^2>}{<p>} \tag{S4}$$

versus

$$P(1|0) \underset{T_i \to \infty}{\longrightarrow} \frac{\sum_{i=1}^{N} T_i \cdot (1-p_i)\cdot p_i}{\sum_{i=1}^{N} T_i \cdot (1-p_i)} = \frac{<p> - <p^2>}{1-<p>} \tag{S5}$$

where T_i is the number of throws player i has taken and S_i is the number of successful throws out of them. The averages are weighted by the number of throws each player made. Here as well, the results coincide only in cases where the p_i's are all the same and hence $<p><p>=<p^2>$. For a large number of players with probabilities

drawn from a uniform distribution between P_{min} and P_{max} (each of the players took equal amount of throws), this result is:

$$P(1|0/1) = P^{+} \mp \frac{P^{-2}}{3 \cdot P^{+}} \tag{S6}$$

where $P^{+} = \frac{P_{max}+P_{min}}{2}$ and $P^{-} = \frac{P_{max}-P_{min}}{2}$. One is referred to figure S1 to see how this calculation works for simulations of two choices of T and N.

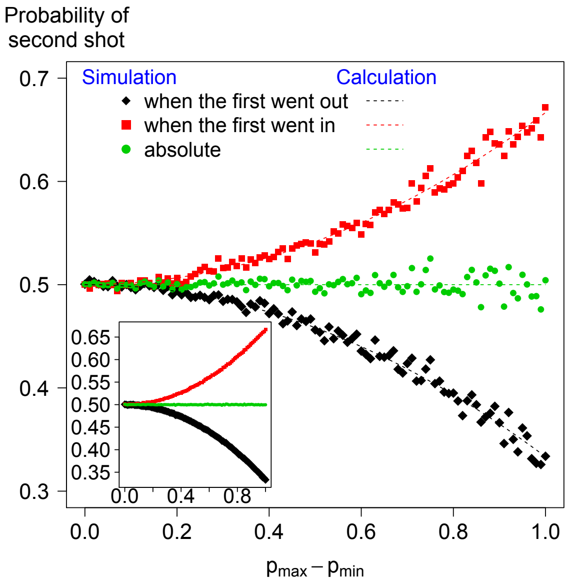

Figure S1. The results of $N = 600$ hypothetical players that shoot 100 pairs of throws . The players have been attributed with probabilities for successful throws that were drawn from a uniform distribution around $P^+ = 0.5$ with varied size (the x axes, $2 \cdot P^-$). The red squares represent the aggregated probability of successful second throw given that the first one was in, the black diamonds represent the same probability given that the first one went out and the green circles represent the absolute probability of successful second throw regardless the outcome of the first throw. The dashed lines are the result of the analytical calculation (equation S6), the agreement between the simulations and the calculation is evident. In the inset 200000 players were considered, each threw 10 throws in order to emphasis the fact that with large number of players, the analytical expression and the simulations agree perfectly even if each player had small number of throws.

2 Supporting figures and tables

Name	Team	Records	$p(1,)$	$p(,1)$	$p(1\|0)$	$p(1\|1)$	z_{NS}	z_{CP}
Dwight Howard	ORL	371	60.92	58.49	55.17	60.62	-0.67	1.04
Kevin Durant	OKC	333	87.39	90.99	85.71	91.75	1.5	1.28
LeBron James	CLE	330	72.12	79.7	82.61	78.57	2.27	-0.82
Dwyane Wade	MIA	297	71.38	79.8	76.47	81.13	2.39	0.9
Amare Stoudemire	PHX	273	74.73	77.66	78.26	77.45	0.8	-0.14
Carmelo Anthony	DEN	264	83.71	82.58	72.09	84.62	-0.35	1.98
Chris Bosh	TOR	262	79.77	80.53	83.02	79.9	0.22	-0.51
Corey Maggette	GSW	253	83.4	84.19	83.33	84.36	0.24	0.17
Dirk Nowitzki	DAL	246	88.62	94.31	92.86	94.5	2.26	0.35
Gerald Wallace	CHA	246	71.95	82.11	78.26	83.62	2.68	0.98
Kobe Bryant	LAL	230	80.43	82.17	82.22	82.16	0.48	-0.01
Brook Lopez	NJN	223	83.41	81.17	86.49	80.11	-0.62	-0.9
Tyreke Evans	SAC	202	74.75	75.74	74.51	76.16	0.23	0.24
Chauncey Billups	DEN	201	89.55	92.54	95.24	92.22	1.05	-0.5
Zach Randolph	MEM	196	77.55	80.1	81.82	79.61	0.62	-0.32

Table S1. Examples of individual data
The top 15 players (ranked by the number of two throws attempts taken) for the 2009/2010 season are listed here along with their individual numbers.

	2005/2006	2006/2007	2007/2008	2008/2009	2009/2010
$\bar{Z}_{NS}^{1,2}$	-0.215	0.101	0.890	2.370	1.688
$Z_{NS}^{1,2}$	-0.360	0.166	0.289	2.316	1.760
$\bar{Z}_{NS}^{2,3}$	1.808	2.534	0.000	0.457	0.401
$Z_{NS}^{2,3}$	1.960	2.460	0.100	0.452	0.189

Table S2. Significance of the non-stationary trend for three free throws sequences Z_{NS}^{12} is the Z value calculated for the first and second throws while Z_{NS}^{23} is for the second and third throws. the values with the $^{-}$ symbol are calculated for the aggregated data. One sees that apart from 2005/2006, first and second throws, the signs of the calculate Z values are positive which indicates that there is a tendency for the increase of the rate of success as the number of trials increase. However, the data is much smaller (see table 1 in main text for actual numbers of throws) and hence the statistical significance of the results is much weaker.

	2005/2006	2006/2007	2007/2008	2008/2009	2009/2010
$\bar{Z}_{CP}^{1,2}$	1.585	2.142	0.568	0.359	1.699
$Z_{CP}^{1,2}$	0.467	0.074	-0.631	0.321	1.585
$\bar{Z}_{CP}^{2,3}$	-0.157	1.258	0.223	0.737	0.291
$Z_{CP}^{2,3}$	-0.320	1.273	-0.349	1.141	0.292

Table S3. Significance of the CP trend for three free throws sequences Similar to table S2 but for the CP trend. Non of the cases here is statistically significant but the majority of the Z's signs are positive, which indicate a hot hand tendency, but once again, the data is much smaller and hence the statistical significance of the results is much weaker.

	2005/2006	2006/2007	2007/2008	2008/2009	2009/2010
% of 1's	23.3	23.5	23.6	23.9	21.5
$Z_{1's}$	-3.4	-3.1	-2.7	-2.1	-6.9
% of 2's	26.9	26.8	27.1	26.4	25.1
$Z_{2's}$	10.1	9.3	10.7	7.1	0.3
% of 3's	28.4	26.1	23.1	28.7	27.9
$Z_{3's}$	2.0	0.7	-1.3	2.4	2.2

Table S4. Number of free throws sets in the 4th quarter The percentage of throws from various types (series of 1,2 and 3 consecutive throws), in the last quarter (excluding overtime) out of the number of throws in the whole game (excluding overtime). Since by pure chance one expects to observe one quarter of the throws to be on the forth quarter with fluctuations following a Binomial distribution with $p = 1/4$, $Z_{1/2/3}$ are calculated as $Z_{1/2/3} \equiv \frac{T^4_{1/2/3} - \frac{T^{ALL}_{1/2/3}}{4}}{\sqrt{\frac{3 \cdot T^{ALL}_{1/2/3}}{16}}}$ where $T^{ALL}_{1/2/3}$ is the number of 1,2 or 3 consecutive throws sets in the whole game and $T^4_{1/2/3}$ is the number in the 4th quarter. One can see that there are more 2 throws sets in the 4 the quarter than expected and less 1 throw sets than expected. Interestingly in the 2009/2010 season the access of 2 throws sets is not significantly, but in the other seasons it definitely is.

	2005/2006	2006/2007	2007/2008	2008/2009	2009/2010
$\bar{P}_{4th}(1)$	0.720	0.733	0.745	0.754	0.749
$\bar{P}_{1st-3rd}(1)$	0.732	0.724	0.745	0.755	0.738
$\bar{P}_{ALL}(1)$	0.725	0.731	0.747	0.755	0.745
$\bar{Z}_{4th}$	1.453	-1.950	0.290	-0.128	-2.105
$\bar{P}_{4th}(,1)$	0.769	0.783	0.790	0.798	0.789
$\bar{P}_{1st-3rd}(,1)$	0.733	0.727	0.742	0.754	0.737
$\bar{P}_{ALL}(,1)$	0.721	0.735	0.747	0.756	0.746
$\bar{Z}_{4th}$	-1.100	-1.318	0.375	0.032	-0.404

Table S5. **4th quarter and overtime vs. 1st-3rd quarters** First four rows refer to the first throw in all types of sets of throws while last four rows refer to the second throw in a two throws sets only. Data is combined from all players (hence the^{-}), but even at this level there is no significant difference between the different parts of the game. $\bar{Z}_{4th}$ are calculated once again with the aid of the hypergeometric distribution: W_{4th} is the total (in all game) number of *successful* throws, B_{4th} is the total (in all game) number of *unsuccessful* throws, S_{4th} is the number of throws taken on the 4th quarter or overtime and w_{4th} is the number of *successful* throws in the 4th quarter and overtime. Apart from 2006/2007 and 2009/2010 where significance is in the 5% region (but not corrected for multiple tests), the performance in the last part of the game is pretty much the same as at the beginning of it in terms of free throws success rates.

	2005/2006	2006/2007	2007/2008	2008/2009	2009/2010
$\bar{P}(1\|0)$	72.65	71.83	72.44	75.55	74.20
Number of records	5308	4930	4725	4466	4581
$\bar{P}(1\|1)$	78.63	79.34	79.21	80.40	79.53
Number of records	13250	13406	13054	13112	13111
$P(1\|0)$	72.94	74.21	73.20	77.13	73.80
Number of individuals	384	391	381	379	383
$P(1\|1)$	75.28	76.96	76.52	77.32	75.84
Number of individuals	407	415	403	402	396

Table S6. **1st-3rd quarters conditional probability (CP)** Equivalent table to table 3 from main text but for throws taken in 1st-3rd quarters only. The trend observed in the whole game is observed here as well - though the number of throws is lower.

	2005/2006	2006/2007	2007/2008	2008/2009	2009/2010
$\bar{P}(1\|0)$	72.03	73.51	73.02	74.84	72.98
Number of records	2492	2420	2265	2071	2128
$\bar{P}(1\|1)$	78.85	79.78	80.15	81.12	79.84
Number of records	6715	6588	6372	6193	5730
$P(1\|0)$	71.77	73.57	70.93	72.37	73.77
Number of individuals	384	402	377	363	378
$P(1\|1)$	76.97	77.03	75.31	76.45	76.31
Number of individuals	412	415	411	400	408

Table S7. 4th quarter and overtime conditional probability (CP) Equivalent table to table 3 from main text but for throws taken in 4th quarter and overtime only. The trend observed in the whole game is observed here as well - though the number of throws is lower.

	2005/2006	2006/2007	2007/2008	2008/2009	2009/2010
$\bar{Z}_{NS}$	12.15	9.32	8.73	10.17	8.92
Z_{NS}	11.51	9.31	8.65	9.65	8.55
$\bar{Z}_{CP}$	8.75	10.77	9.53	6.89	7.52
Z_{CP}	2.29	3.57	2.83	0.64	1.65
q_{CP}	2.2e-02	3.6e-04	4.7e-03	5.2e-01	9.9e-02

Table S8. Statistical significance of the trends observed in the 1st-3rd quarters

	2005/2006	2006/2007	2007/2008	2008/2009	2009/2010
$\bar{Z}_{NS}$	6.38	7.76	6.93	7.07	7.38
Z_{NS}	6.93	8.23	5.95	5.89	7.24
$\bar{Z}_{CP}$	6.91	6.38	7.06	6.13	6.52
Z_{CP}	2.14	1.91	3	2.53	2.09
q_{CP}	3.2e-02	5.6e-02	2.7e-03	1.1e-02	3.7e-02

Table S9. Statistical significance of the trends observed in the 4th quarter and overtime
